# Life expectancy of *Anopheles funestus* is double that of *Anopheles arabiensis* in southeast Tanzania based on mark-release-recapture method

**DOI:** 10.1038/s41598-023-42761-3

**Published:** 2023-09-22

**Authors:** Watson Ntabaliba, Laura Vavassori, Caleb Stica, Noel Makungwa, Olukayode G. Odufuwa, Johnson Kyeba Swai, Ruth Lekundayo, Sarah Moore

**Affiliations:** 1 Vector Control Product Testing Unit (VCPTU), Ifakara Health Institute, Environmental Health, and Ecological Sciences, P.O. Box 74, Bagamoyo, Tanzania; 2 Vector Biology Unit, Department of Epidemiology and Public Health, Swiss Tropical and Public Health Institute, Kreuzstrasse 2, 4123 Allschwil, Switzerland; 3 University of Basel, Petersplatz 1, 4001 Basel, Switzerland; 4 Queensland University of Technology, Brisbane, Australia; 5 MRC International Statistics and Epidemiology Group, Faculty of Epidemiology and Population Health London School of Hygiene and Tropical Medicine, London, UK; 6 Nelson Mandela African Institute of Science and Technology, Tengeru Arusha, Tanzania

**Keywords:** Ecology, Ecology

## Abstract

*Anopheles arabiensis* and *Anopheles funestus *sensu stricto mosquitoes are major East African malaria vectors. Understanding their dispersal and population structure is critical for developing effective malaria control tools. Three mark-release-recapture (MRR) experiments were conducted for 51 nights to assess daily survival and flight range of *An. arabiensis* and *An. funestus* mosquitoes in south-eastern, Tanzania. Mosquitoes were marked with a fluorescent dye as they emerged from breeding sites via a self-marking device. Mosquitoes were collected indoors and outdoors using human landing catches (HLC) and Centers for Disease Control and Prevention light traps (CDC-LT). In total, 4210 *An. arabiensis* and *An. funestus* were collected with 316 (7.5%) marked and recaptured (MR). Daily mean MR was 6.8, standard deviation (SD ± 7.6) for *An. arabiensis* and 8.9 (SD ± 8.3) for *An. funestus.* Probability of daily survival was 0.76 for *An. arabiensis* and 0.86 for *An. funestus* translating into average life expectancy of 3.6 days for *An. arabiensis* and 6.5 days for *An. funestus*. Dispersal distance was 654 m for *An. arabiensis* and 510 m for *An. funestus*. *An. funestus* life expectancy was substantially longer than that of *An. arabiensis*. The MRR method described here could be routinely utilized when evaluating the impact of new vector control tools on mosquito survival.

## Introduction

Mainland Tanzania has been classified by World Health Organization (WHO) as a country with high malaria burden that requires targeted application of malaria control tools^1^. Both malaria cases and malaria deaths have increased in recent years due to population growth^2^, insufficient coverage of vector control tools^3^ and to some extent the increase of malaria vector resistance to insecticides used in vector control^4^. Malaria transmission is highly heterogeneous^5^ due to geographical differences including altitude, urbanization and vegetation^6^. For these reasons, the country has stratified malaria control deploying tools based on the intensity of malaria transmission^7^ as a response to the WHO high burden to high impact (HBHI) strategy^8^. Much of the finer scale (district) differences in malaria transmission intensity is attributable to the vector species composition in that area. To maximize resources, the deployment of malaria vector control needs to be targeted against those vectors that transmit most of the disease.

In south-eastern Tanzania, malaria transmission is mediated by *Anopheles funestus *sensu stricto and *Anopheles arabiensis* that differ markedly in their vectoral capacity^9^. *An. funestus* feeds on humans primarily indoors and late at night^10^ while *An. arabiensis* feeds on humans and cattle indoors or outdoors^11^. These differences in ecology result in different man-vector contact that to some extent explains their differing vectoral capacity^12^.

Adult survival is a critical component of vectoral capacity because adult females must survive the extrinsic incubation period of *Plasmodium* before they can transmit pathogens^13,14^. Vector control tools reduce mosquito infectivity rate^15^, density and daily survival, with the later having the greatest impact on malaria transmission^16,17^. In addition, the use of insecticides may also disproportionally increase mortality among older mosquitoes and can partially reduce the impact of insecticide resistance^18^. Other than old age, several other factors such as disease, predation or environmental factors contribute to mosquito mortality^19^. Therefore, it can be argued that vector population age structure is a more valid metric^20^ than mosquito density as an outcome in entomological trials of vector control tools, as has been elegantly demonstrated in early trials of Insecticide Treated Nets (ITNs)^21^.

Dispersal of *Anopheles* mosquito range between few meters to several kilometres depending on resource availability i.e. larval habitats, sugar and blood hosts^22^ and species-specific environmental adaptability^23^. The range has been demonstrated to be bigger in rural areas, with relatively higher mobility compared to urban areas^24^. Laboratory-reared *Anopheles* have been found to disperse more than wild mosquitoes, possibly due to the presence of a memorised home range^25^. *Anopheles* mosquitoes actively disperse only or primarily during part of their gonotrophic cycle^26^. This is epidemiologically important as it influences the extent of malaria parasite acquisition and distribution that leads to transmission by female mosquitoes.

Mosquito population parameters can be estimated through a number of morphological, biochemical, genetic and spectroscopic methods, each of which has limitations in reliability, specificity, validation, and requires high cost or the need for an expert’s technical ability^27^. MRR is a technique where mosquitoes collected in the wild or reared in the laboratory, are marked, released then recaptured at a given distance and time interval from the releasing point^28–30^. MRR is an effective and low cost means of investigating adult mosquito dispersal and survivorship that can be utilized in most settings and has been evaluated against multiple species^31^. MRR experiments include a single mark and release of mosquitoes, followed by one or repeated recaptures^32^.

This study investigated the survival and dispersal capabilities of *An. arabiensis* and *An. funestus* in Ikungua village by marking mosquitoes as they emerged from their breeding sites^33^. Using mosquitoes as they emerge means that the technique is more reflective of natural dispersal and ethically less challenging as additional mosquitoes are not introduced and wild mosquitoes from the environment are recaptured. This data is needed to inform mathematical models used to optimize the selection of malaria control tools^7^. Furthermore, knowledge of survival and dispersal of malaria vectors is critical for planning, evaluating and implementing new tools which are intended to interrupt the pathogen’s transmission^34^.

## Results

### Mosquito release

A total of 4210 mosquitoes (both *An. arabiensis* and An. *funestus*) were marked and released into the wild mosquito population (with correction factor of 86% marking efficiency^33^), over three separate releases and 17 days follow up for each release.

### Mosquito recapture

A total of 13,359 (*An. arabiensis* and *An. funestus*) marked and unmarked mosquitoes were collected and morphologically identified as *An. gambiae s.l.* (7260) and *An. funestus* s.l. (6099). Polymerase chain reaction (PCR) showed all the *An. gambiae* s.l. tested to be *An. arabiensis* (50/50 succesful amplifications). Furthermore, for *An. funestus* group, 92% (46/50 successful amplifications) showed to be *An. funestus* s.s whilst 8% (4/50) of the samples did not amplify. Recapture rate was 3% (n = 138) and 4% (n = 178) with a daily average of 6.8 (SD ± 7.6) for *An. arabiensis* and 9 (SD ± 8) for *An. Funestus*, respectively. Daily recapture declined over time from 16 (5.3%) on the first day to 1 (0.3%) on the 15th day after each release. There were few recaptured male mosquitoes, 12 *An. gambiae s.l.* and 4 *An. funestus s.l.* because we used recapture methods that targeted host seeking female mosquitoes. Males were excluded from the analysis.

### Daily survival probability

**Figure 1:**
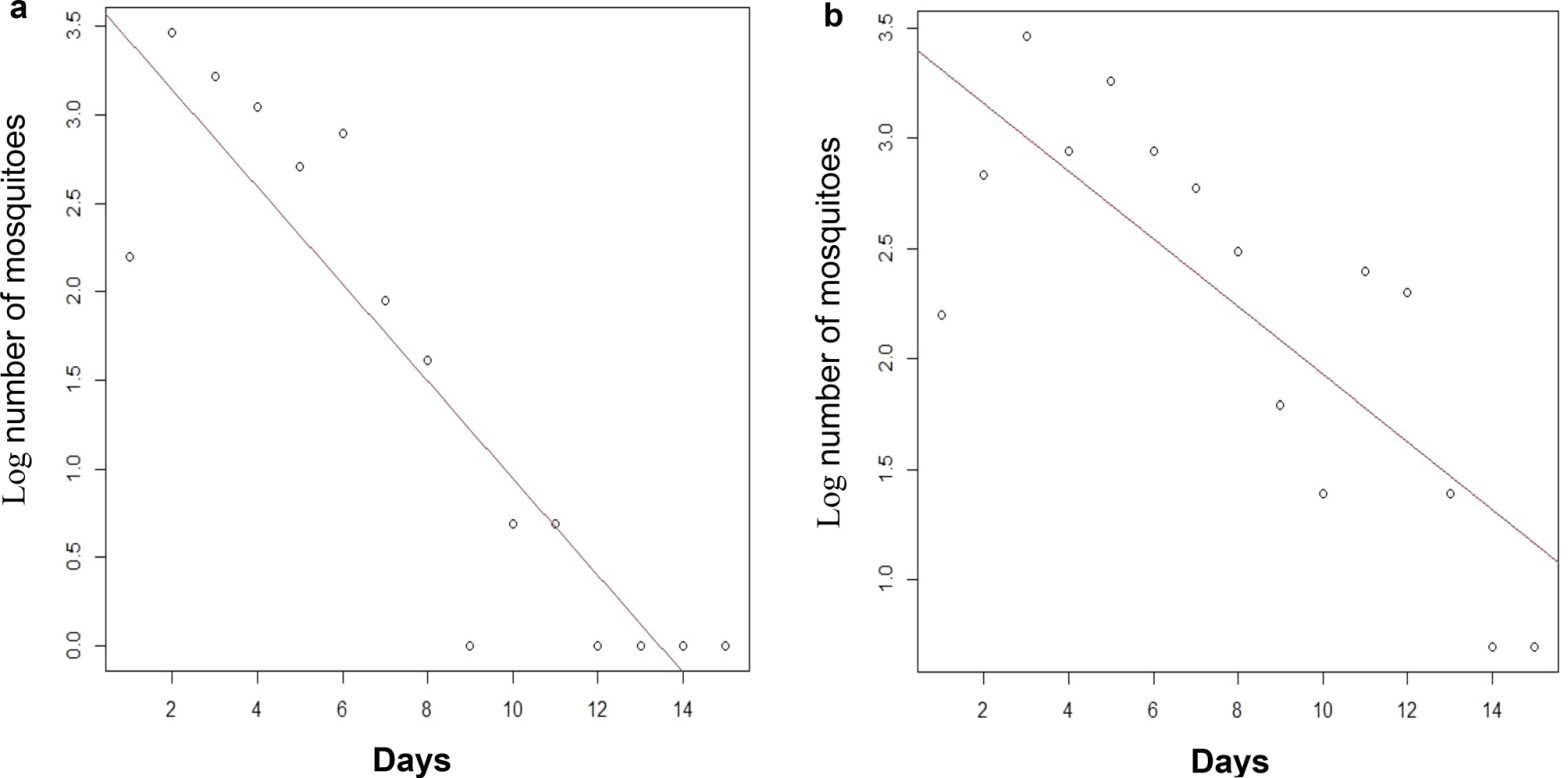
Probability of survival trends for *An. arabiensis* (**a**) and *An. funestus* (**b**) over a period of 2 weeks. Overall, for both species’ survival reduced over time but, *An. funestus* seemed to live longer than *An. arabiensis* since recapture of marked and released mosquitoes extended beyond the 15th day.

The daily survival probability was 0.76 for *An. arabiensis* and 0.86 for *An. funestus* which equates to a life expectancy of 3.64 and 6.51 days, respectively (Fig. 1).

### Average dispersal distance

**Figure 2:**
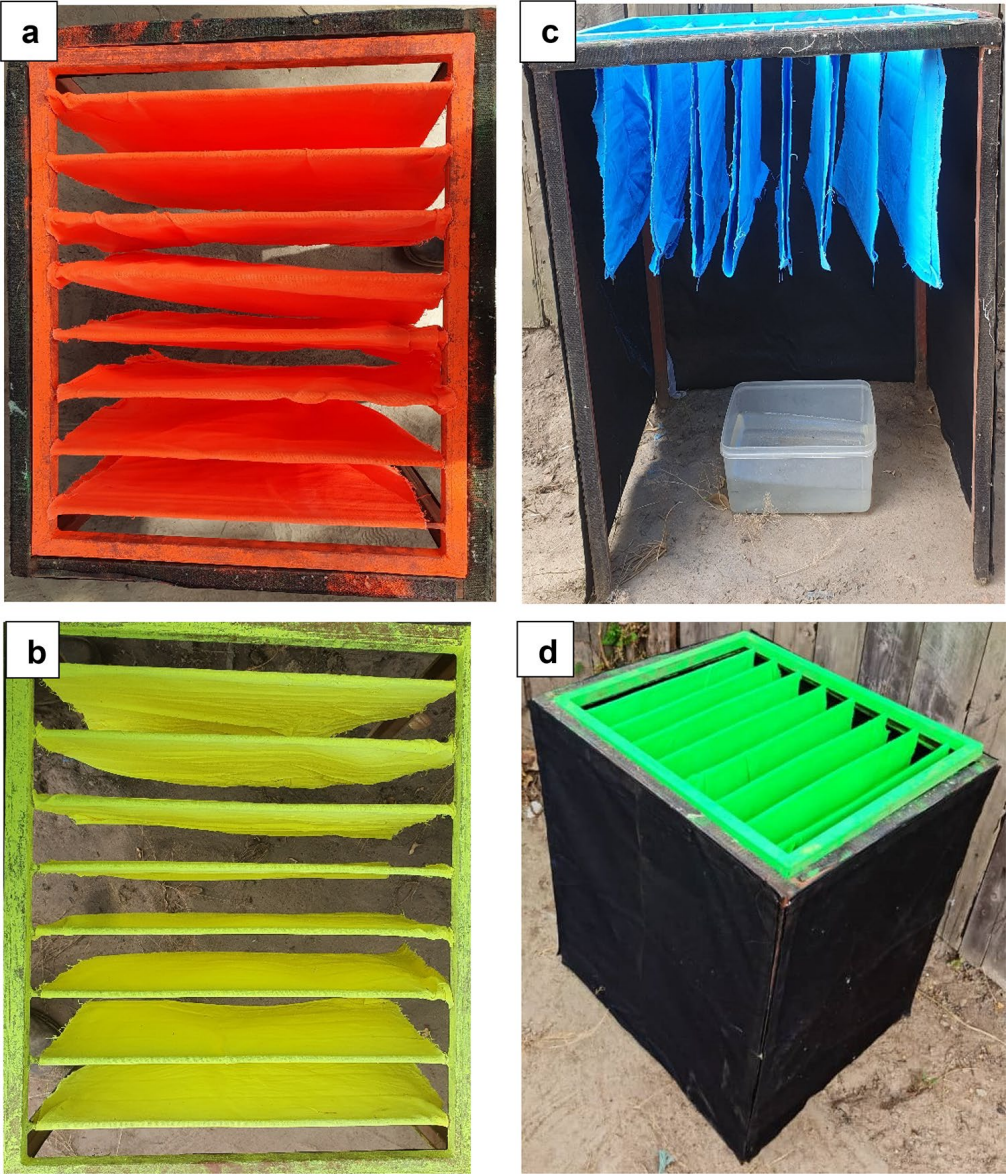
The self-marking unit used previously in Saddler et al., (**a**,**b**) are respectively impregnated clothes with orange and yellow fluorescent dye. (**c**) The frame attached black cloth into a Velcro with pupae bowl and blue fluorescent dye. (**d**) The unit set up fully closed for release experiment and green fluorescent dye.

The average dispersal distance of marked mosquitoes was 654 m (95% CI 543–763) for *An. arabiensis* and 510 m (95% CI 450–570) metres for *An. funestus*. The probability of capturing a marked mosquito declined over distance (Table 1). The majority (95.6%) of *An. arabiensis* and a lower proportion (75.8%) of *An. funestus* were recaptured within 500 m of the releasing point. Fewer mosquitoes were recaptured in the third annulus compared to the fourth annulus; 0.7% versus 3.6% for *An. arabiensis* and 2.2% versus 21.9% for *An. funestus*. There was a similarity between maximum recapture for *An. arabiensis* (20.5%) and *An. funestus* (20.0%) at 6 days after mosquito release, while the rate dropped dramatically from day 7 to 15 with 1.6% *An. arabiensis* and 6.4% *An. funestus* recaptured (Fig. 2).

**Table 1:** Number of *An*. *arabiensis* and *An. funestus* recaptured by distance from the release point in Ikungua village.

Annulus	Distance from release (m)	*An. Arabiensis * n (%)	*An. Funestus * n (%)
1st	215	78 (56.5)	71 (39.9)
2nd	430	54 (39.1)	64 (35.9)
3rd	645	1 (0.7)	4 (0.02)
4th	860	5 (3.6)	39 (21.9)
Total		138 (100)	178 (100)

### Estimates of population size

Population size for *An. arabiensis* was estimated as 101,886 mosquitoes while that of *An. funestus* was estimated as 78,991 mosquitoes. There were approximately 443 *An. arabiensis* and 343 *An. funestus* per hectare of the study area.

## Discussion

This study was designed to investigate the mobility and life expectancy of two major malaria vector populations in south-eastern, Tanzania. Mosquito longevity is a critical aspect in transmission of *Plasmodium falciparum* that requires > 12 days to be infective^17^. The longer lifespan of *An. funestus* may have therefore contributed to its relative efficiency as a vector compared to *An. arabiensis* despite its comparatively low abundance. More focused attention is needed on the control of *An. funestus* due to its efficiency in transmitting malaria^35^.

### Survival

Data from this study demonstrates that *An. funestus* survives longer, and has a 10% higher daily survival probability than *An. arabiensis*. These findings corroborate with earlier studies done in the Kilombero valley^36,37^ as well as other studies from Tanzania^38^ and West Africa^39^ that reported *An. funestus* having higher survival rates and lower mortality than *An. arabiensis.* This higher survival may be as a result of its adaptation to readily available human hosts and reported resistance^9,35,40^ against pyrethroid insecticides used in ITNs implemented for malaria control in this area^41^. These adaptations (endophily and anthrophily), also make it extremely vulnerable to control with core tools using insecticides to which *An. funestus* is susceptible. It should be noted that *An. arabiensis* in the region are also highly pyrethroid-resistant^42^. However, *An. funestus* has been shown to be more efficient in malaria transmission than *An. arabiensis* which may be related to differences in resistance or behaviour^35^ whereas laboratory studies have reported similar survivorship for *An. arabiensis*^43,44^ and *An. funestus*^43^ under a standard culturing environment.

Although, the estimated survival probability of *An. funestus* in this study (0.84) is 2% lower than the study conducted in the same area in the 1990’s (0.86)^36^ given the higher coverage (> 50%) of ITNs^45^ and other insecticide-based interventions than in the previous years, we hypothesize that pyrethroid resistance may be the reason for the maintained life expectancy of the species.

Changes in mosquito population survival may also be evaluated through dissecting the ovaries^46^ to measure the number of gonotrophic cycles that mosquitoes have undergone^47^ or the proportion of the proportion that have ever laid eggs^48^. The Detinova dissection technique is straightforward but requires dedicated technical staff, but very few people are skilled enough to routinely carry out the Polovodova technique^49^. Other age grading techniques include mid infra-red spectroscopy^50^, near-infra red spectroscopy^51^ as well as molecular methods such as transcription methods^52^. However, these more recent methods are still in development and are not used routinely^53^.

### Dispersal

The dispersal distance of *An. arabiensis* and *An. funestus* was determined by measuring the distance travelled between the releasing point and the recapture house. *An. arabiensis* was found to have a similar dispersal distance to *An. funestus.* Similarly, Saddler et al.^33^ using the same MRR method, found the mean dispersal distance of *An. arabiensis* in Bagamoyo to be 579 m (95% CI 521–636), which is similar to that obtained for *An. arabiensis* in the current study. However, other researchers have reported higher dispersal distances. Wada et al. recorded individual mosquitoes travelled 5100 m within a day of being released^54^. Thompson et al.^24^ recorded *An. gambiae* up to 1400 m from the releasing site. But, Midega et al*.* found no difference in the mean dispersal distance between *An. funestus* and *An. arabiensis* along the Kenyan coast^55^.

Differences in dispersal distances may be due to varying geographical terrains^56^, density and location of human hosts, availability of sugar sources, oviposition, breeding and resting sites^22^ as well as environmental factors like prevailing wind direction, humidity and temperature^55^. During the course of the study, one of the houses located in the 4th annulus registered an unlikely large number of recaptured *An. funestus* (17.4%). Further, investigations showed that the house was close to a seasonal breeding site and had twice (6 members) the average number of people compared to the other households (2.8 members). There is existing evidence supporting the occurrence of higher densities of malaria vectors in households located near breeding sites^57^ and in those with higher number of individuals due to increased levels of carbon dioxide, a long range attractant of host-seeking mosquitoes from the presence of more residents^58,59^.

In the current study, females of both species (*An. arabiensis* and *An. funestus*) dispersed and recaptured at the furthest house were found 860 m from releasing site in the fourth annulus, but only a small proportion of mosquitoes (12.3%) reached this distance. Additional studies are needed with sampling more evenly distributed mosquitoes and carried out throughout the year to better understand the drivers of dispersal including population biomass and location of breeding sites in the study sites.

### Release

Most MRR studies, mark adult mosquitoes that have been collected from the local area and release them at a central point^31^. The abundance of mosquitoes in MRR experiments is likely to be higher in houses close to the releasing point as found in our study because of the short distance between the houses and the releasing point.

In earlier investigations in Kikulukutu village, south-eastern, Tanzania^60^, adults of unknown age who have probably completed part of their gonotrophic cycle were released. When comparing aging mosquitoes captured and released in some studies^60^, results suggest the use of younger mosquitoes (pupa and larvae) as they are more likely to survive longer which increases the chance of them being recaptured^61^. Use of young mosquitoes of a known age also allows determination of mosquito life span.

### Recapture

Most marked mosquitoes were recaptured in neighbouring houses several hours later after being released. The house closest to the releasing site which was 130 m away, had more *An. funestus* recaptured compared to *An. arabiensis*. This could be due to anthropophilic and endophilic nature of *An. funestus*^62^. Although widely used, MRR experiments are often limited by recapture rates below 5% of those released^60,63^. The overall recapture rate of 7.5% observed in the current study is double the average, 3% (1–9.5%) reported in the literature for *Anopheles*^31^. Fewer houses were located in the third annulus may explain why fewer mosquitoes were recaptured in the third annulus relative to the fourth annulus. Typically, more recaptures were made near the release point (first annuli) for both species and decreased as one moves further away from the releasing point. Therefore, greater sampling effort is required at greater distance from the releasing point.

The limitations to this study were refusal of some households to allow mosquito collections, resulting in unequal distribution of houses in each annulus. This was minimized by standardizing the study regions to accommodate all four annuli. Also, to simulate the natural environment, the release point was close to the natural breeding site located on the village periphery rather than centre in the study area. We did not collect data on the biomass in all houses or the location of all breeding sites in the village. A more comprehensive mapping effort at the beginning of the study would have allowed us to better understand mosquito dispersal. Another limitation was failure to get resistance profile of the two-mosquito species from the study area during the study however *An. Arabiensis* has been shown to be resistant to pyrethroid^42^.

In further studies of MRR the use of indoor resting collections and mosquito abdominal status is recommended to measure if the dyes affect the ability of mosquitoes to feed. The validity of the MRR method rests on the assumption that marked individuals behave in every respect as the unmarked ones (wild), and that both marked and unmarked mix together in a homogenous, random way. MRR includes marking mosquitoes with a fluorescent dye, a procedure that has been reported by many investigators to not affect the survival and dispersal behaviour of mosquitoes provided it is applied correctly^64^. The marking method was investigated during the development of the MRR method used here and found not to affect survival^33^ but it is not known if it can affect flight or predator response to marked insects.

## Conclusion

MRR used as mosquitoes emerge from breeding sites is a simple and cost-effective method for measuring the dispersal and survival of mosquitoes. It can be deployed as part of routine entomological collections when evaluating vector control tools with active ingredients that that are designed to overcome resistance to existing classes of insecticides and consequently reduce mosquito population life expectancy when deployed at scale. This study has demonstrated that *An. funestus* has a substantially longer life expectancy than *An. arabiensis* in this setting, which may partially explain the greater efficiency of *An. funestus* in malaria transmission.

## Methods

### Study area

Three experimental phases of MRR survey were performed during the study, which was conducted at the end of the rainy season between September and October 2020 in Ikungua village in South-eastern, Tanzania^65^. The village had 347 houses and 984 inhabitants over 36 hectares. The village is surrounded by forests, water bodies, and agricultural areas. The temperature ranged from 23 to 32 °C during the study and the annual rainfall ranges from 1200 to 1800 ml. Residents are subsistence farmers, growing bananas and millet in the hillsides whilst growing rice in the valleys through an irrigation system, which provides breeding sites for malaria vectors. *An*. *gambiae* s.s populations have significantly declined in the study area^35^ leaving *An. arabiensis* and *An. funestus* as the main vectors with *An. funestus* mediating majority of the infections even though it is present in lower densities than *An*. *arabiensis*^9^. House structures in the village allow indoor entry through opened eaves, mud walls, thatch roofs, and doors not covering the whole entrance as well as windows^66^. National malaria control is implemented through Insecticide Treated Nets (ITNs) in the study area. Although, a field study of an indoor residual spray product was implemented in the villages shortly (about 1 year) before the study.

### Mosquito preparation

Wild *An. arabiensis* and *An. funestus* pupae and larvae stage 2–4 were collected from multiple natural breeding ponds and puddles located within a one thousand meters radius from the releasing point using a larval dipper and one-millimetre bulb pipette. The colony was maintained in a field laboratory with 300 larvae per bowl reared at 25 ± 7 °C temperature and 40–99% relative humidity. These pupae and stage 4 larvae were maintained in a plastic bowl with some water and placed underneath the marking trap near a shelter in releasing area. After emergence, adult mosquitoes on their first flight out to seek for food and mate pass through pigment impregnated onto cloth strips where they would be marked with dye pigments.

**Figure 3:**
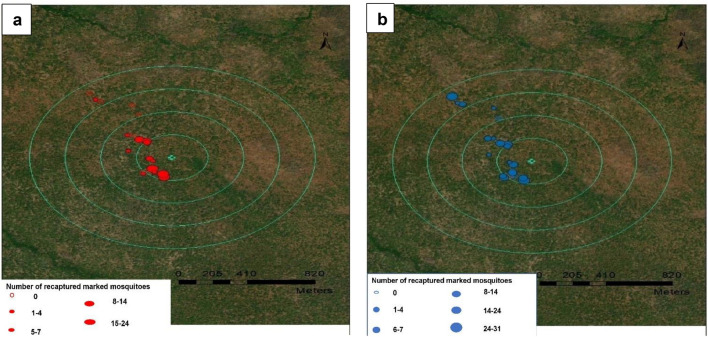
Distribution of mosquito collection houses in four annuli with the dot size point indicating estimated mosquito collected in the house. (**a**) Presents *An. arabiensis* with red marker. (**b**) Presents *An. funestus* with blue marker. Base maps were provided by Open source QGIS^71^.

Each release was conducted for 5 days consecutively with daily average of 281 pupae/larvae placed under the trap each day at 18:00 h. Recapture was conducted each night of the release and for 12 days after the last release, total 17 nights of recapture. There was then a wash out period of a further 3 days before the next round was conducted. In total, three rounds of MRR were conducted. The total mosquitoes released were: 794 in 1st, 2025 in 2nd, and 1435 in the 3rd release. A single releasing point, close to the mosquitoes’ natural breeding site was used for all of the releases (Fig. 3).

### Marking unit

A self-marking unit^33^ was used to mark *An. arabiensis* and *An. funestus* in the field experiments (Fig. 2). There were five marking grids with a different colour used each day: pink, yellow, blue, orange, and green to distinguish each of the 5 days of release (Wtrcsv, Shenzhen Guang Chen Technology Co., China). The pigments have been shown to not affect mosquito survival^33^.

### Recapture

Mosquitoes were recaptured from 20 houses located in four distance groups (annuli) from the releasing site; 215, 430, 645, and 860 m. Mosquito collection started on the day of the release and was conducted for 17 consecutive days between 18:00 h and 06:00 h using CDC-LT beside human-occupied bed net^67^ in 14 houses and paired indoor and outdoor HLC conducted by adult male volunteers^29^ in six houses. Collected mosquitoes were examined morphologically and identified following taxonomic keys^68^ and examined for fluorescent pigment using an ultraviolet light torch (21 LED 395 nm) and 10 × dissection microscope (ZEISS industrial metrology, Germany). A total number of 100 mosquitoes, 50 *An. gambiae* s.l. and 50 *An. funestus* s.l. were packed in Eppendorf tubed on silica and taken to the IHI Ifakara laboratory for PCR speciation^69,70^.

### Analysis

Mean distance travelled (MDT), was used to describe the distance travelled by the estimate the dispersal of the released mosquitoes. This method estimates movement of adult mosquitoes against the radius of the experimental area^72^, with the assumption of having different densities of traps in different annuli^73^. As the area of the annuli increases with greater distance from the release site a correction factor (CF) for each annulus was estimated by dividing the area of the annulus by the sum of the area of all four annuli and multiplying the result by the total number of traps in the specific annuli^74^. Area of each annulus was calculated using half of the distance from the releasing site as the radius. Then, the number of mosquitoes recaptured in each annulus was divided by the total number of traps in the annuli and multiplied by the correction factor of the annulus to get estimated recapture (ER). Using the ER, cumulative estimated recapture (CER) was calculated. The MDT was then calculated as the sum of the product of ER and radius of the annuli over the total CER.

Survival was estimated with linear regression approach defined by Buonaccorsi et al.^75^ with recaptured mosquitoes adjusted in the model and for the average life expectancy was calculated according to Niebylski and Craig^76^.

Population size was estimated using the Lincoln Index (Eq. 1). The Fisher–Ford and Lincoln Index*,* are simple methods of estimating population size^29^ in mark-release experiments. The method assumes that: (1) marked and wild mosquitoes mix homogeneously immediately after release in a random way, (2) random dispersal of the marked and wild population without loss or gain in the population, and (3) there is a constant mortality rate among released mosquitoes. Using this method, estimates of total population size (P) are determined by the numbers of mark-released mosquitoes (*a*), the number captured on the subsequent occasion (*n*), and the number of those recaptured which had been marked (*r*), when *r* is greater than 20. In this study, it was assumed half of the marked-released mosquitoes were *An. arabiensis* and half were *An. funestus*. The analysis was done using R statistical software v4.1.1^77^

1 $$P = \frac{an}{r}$$

P = Population size, a = Mark-released mosquitoes, n = Total captured mosquitoes, r = Mark-recaptured mosquitoes.

Equation (1) showing population size calculation.

### Ethical approval

Ethical approval was granted by the Institutional Review Board of the Ifakara Health Institute (IHI) and Tanzanian National Institute of Medical Research (NIMR) (NIMR/HQ/R.8a/Vol. IX/2894). Written informed consent was obtained from household heads of the twenty houses selected for mosquito collection and from volunteers who performed HLC. The volunteers were provided doxycycline prophylaxis as per Tanzania Ministry of Health guidelines^78^ and were medically supervised^79^. I confirm that the recommended guidelines from the ministry of health were properly followed.

## Data Availability

Datasets shall be provided based on reasonable requests from the corresponding author.
